# Supplementary material for: Hyaluronic acid: Function and location in the urothelial barrier for bladder pain syndrome/interstitial cystitis, an in vitro study
Source: PLoS One. 2025 Feb 26;20(2):e0318277. doi: 10.1371/journal.pone.0318277 (PMC11864510; doi:10.1371/journal.pone.0318277)
Supplement: Attachment — Submitted filename: PlosOne_revision.docx [file pone.0318277.s002.docx]

In this manuscript, van Ginkel at al show the localization of hyaluronic acid in tissue specimen of normal human and porcine urothelium, as well as evaluate different GAG replenishment therapies, used in clinical practice for treating interstitial cystitis, in in vitro model. Though the manuscript presents novel information and is potentially of interest, it presents with some flaws and it might lead to premature conclusions. Some additional experiments are required as well as improvements in the manuscript before it would be suitable for publication.

1. The third objective was to evaluate GAG therapies on an inflamed in vitro model, however, treating cells with PS does not lead to a substantial inflammation as seen in patients with IC/BPS. An in vitro model that employes PS together with another agent, such as LPS, TNFa or H2O2 should be used to better explain the effects of GAG therapies. I would strongly encourage the authors to add these additional experiments.
2. Methods: in all descriptions the number of biological replicates and technical replicates (number of independent experiments) is missing. Please add this information. Please also add how many human and porcine biopsies were used.
3. Why was the in vitro cell culture not prepared from human tissue since it was available?
4. Why did you decide to use different differentiation protocols for different experiments? If a very high TEER was achieved after 3 weeks of culture, why these cells were not used in subsequent experiments?
5. Why did you only evaluate gene expression of tight junctions? These should also be evaluated using ICH or IF, since gene expression does not always reflect the protein expression nor the distribution of tight junctions.
6. Statistical analysis: which post-hoc test was used to assess the differences between groups? Please add.
7. You state that HA was most abundantly present in the basal membrane of the urothelium, however, this should be labeled to justify your findings.
8. In figures with ICH, the scale bars are missing.
9. All figures should include information on how many biological/technical replicates, the labeling or other analysis was performed.
10. All figures should include an explanation of what we see: is it mean or median+/- SD, SEM?
11. Figure 3a: there is no legend explaining what individual lines represent.
12. Figure 4: please add what does the dotted line at y=1 indicate. Please also change graphs to show each individual value of a biological/technical replicate. Were there no significant results in this graph?
13. Why would GAG therapy increase inflammatory markers? I am not sure that the explanation about acute phase response is enough. If this is correct, then the expression of other markers that indicate resolution of inflammation or tissue fibrosis should also be evaluated.
14. If the integrity of urothelium recovers anyway after 24h then what is the point of treatment with GAG replenishment therapy? As written before, additional experiments with a more prominent inducement of inflammation should be employed.
15. For in vitro differentiation of NPU cells achieving high TEER values after 3 weeks, and resemblance with normal human urothelium, the protocol has already been described in literature. Please add the following citations:

<https://link.springer.com/article/10.1007/s00418-014-1265-3>

<https://www.sciencedirect.com/science/article/pii/S0887233317302242?via%3Dihub>

16. Why would be beneficial to generate a stronger urothelial barrier in healthy urothelium? But then in damaged urothelium, this therapy fails to improve the barrier? You are trying to replicate IC/BPS in which there is a leaky urothelium, do you implicate that this kind of therapy should not be used in IC/BPS? How would your findings translate into clinical settings?
17. Why would a mild inflammatory response increase impermeability of urothelium? In IC/BPS, there is an inflammatory response with increased permeability – inflammatory mediators are thought to further increase the damage of urothelial cells and further increase permeability though a positive feedback mechanism? How do you comment on this?
